# Ovarian function after the use of various hemostatic techniques during treatment for endometrioma: protocol for a randomized clinical trial

**DOI:** 10.1186/s13063-019-3524-z

**Published:** 2019-07-09

**Authors:** Raquel Silveira da Cunha Araujo, Sabina Bastos Maia, Clara Micalli Ferruzzi Baracat, Moisés Diogo Lima, Eduardo Sérgio Sousa Soares, Helizabet Salomão Abdalla Ayroza Ribeiro, Paulo Augusto Ayroza Galvão Ribeiro

**Affiliations:** 1 Department of Obstetrics and Gynecology, Lauro Wanderley University Hospital, Federal University of Paraíba, João Pessoa, Paraíba Brazil; 2 Gynecological Endoscopy and Endometriosis Division, Department of Obstetrics and Gynecology, Santa Casa de São Paulo School of Medical Science, São Paulo, São Paulo Brazil

**Keywords:** Endometrioma, Laparoscopy, Ovarian reserve, Anti-Mullerian hormone

## Abstract

**Background:**

Laparoscopic cystectomy is currently considered the gold standard for the treatment of ovarian endometrioma, resulting in an improvement in symptoms, a lower recurrence rate, and a higher pregnancy rate among infertile patients. However, this treatment is not free from risk, since it is associated with a reduction in ovarian reserve. There is still controversy in the literature regarding whether the cause of the reduction in ovarian reserve is due to damage caused by the coagulation energy during hemostasis or whether the procedure itself is the cause of the damage irrespective of the hemostatic method used. The aim of this study is to compare the effects of different hemostatic methods on the ovarian function of women subjected to laparoscopic surgery for ovarian endometrioma.

**Methods:**

An open-label randomized clinical trial to be conducted at the Lauro Wanderley University Hospital between December 2017 and August 2020. Eighty-four patients will be randomly allocated to three groups according to the hemostatic technique used during laparoscopic surgery for ovarian endometrioma: bipolar coagulation; laparoscopic suture; and hemostatic matrix. Ovarian function will be assessed by serum anti-Müllerian hormone measurement and by performing an antral follicle count using ultrasound before surgery and one, three, and six months after surgery. The internal review board of the Medical Sciences Center, Federal University of Paraíba approved the study protocol under reference CAAE 71621717.9.0000.8069.

**Discussion:**

Bearing in mind the need for more randomized clinical trials to clarify this issue, we hope to contribute with data that will determine whether there is any difference between hemostatic methods despite the rational use of bipolar energy or whether the procedure itself explains the ovarian damage irrespective of the hemostatic technique used.

**Trial registration:**

ClinicalTrials.gov, NTC03430609. Registered on XX.10/31/2017.

ISRCTN Registry, ISRCTN11469394. Registered on XX.17/12/2017.

Unique Protocol ID: U1111–1203-2508.

## Background

Ovarian endometriomas are present in 17–44% of women with pelvic pain and infertility. There is considerable controversy regarding aspects that range from variations in the physiopathology of endometriomas to the different forms of treatment [1, 2]. Treatment may consist of conservative management or surgery. Surgical procedures include aspiration of the chocolate-colored fluid from the endometrioma, drainage of the cyst followed by bipolar coagulation, fenestration and laser ablation, cystectomy with complete resection of the endometrioma wall, or even oophorectomy in exceptional cases [3–5].

Laparoscopic cystectomy appears to be the ideal treatment option when surgery is required. This procedure offers favorable results in terms of improving pelvic pain and reducing recurrence rates of the endometrioma [4, 6, 7]. Nevertheless, the technique is not without risk and may result in damage to ovarian function, either from the inadvertent removal of healthy ovarian parenchyma or as a consequence of the thermal effect of coagulation on bleeding points [8–13].

Findings that the surgical treatment of endometriomas can damage ovarian function led to several studies being conducted, resulting in hundreds of publications on the subject. However, no definitive answers have been found. Whereas some studies reached the conclusion that surgery is responsible for ovarian damage [4, 7, 11, 14–26], others reported that the mere fact that the patient had an endometrioma justifies the damage, since the disease in itself constitutes a major cause [27–35].

In relation to the surgical technique used, the majority of investigators agree that the use of bipolar coagulation is responsible for a decrease in ovarian function when this technique is compared with hemostasis with suture or with the use of hemostatic agents [9–13]. Nevertheless, the available evidence is inconclusive, since some studies have failed to find any significant decrease in ovarian function [6, 36–39].

Ata et al. recently conducted a meta-analysis that included six studies, four of which consisted of randomized clinical trials (RCT). Of these, two included only patients with bilateral endometriomas, a situation that is known to be associated with an increased risk of impaired ovarian function compared to unilateral endometriomas, irrespective of the hemostatic technique. Furthermore, the investigators concluded that the quality of the evidence in the studies analyzed in that meta-analysis was moderate and that it would be advisable to avoid the use of bipolar energy whenever possible. In view of the small magnitude of the effect, the possible side effects and the additional cost of hemostatic agents, it is not possible to recommend substituting bipolar coagulation with another method. Indeed, those investigators suggested that further RCTs should be conducted to compare different hemostatic techniques [13].

Another meta-analysis included some widely heterogenous studies that compared laparoscopy with laparotomy, used different methods for the evaluation of ovarian function, and included cases of bilateral endometriomas. The authors suggested that new clinical trials should be conducted to compare different hemostatic methods in order to provide further clarification [12].

Notably, none of the studies in which bipolar coagulation was evaluated provided details on how the bipolar energy was used, stating only the voltage that was applied. Therefore, there are no precise data on how many points were coagulated or for how long cauterization was performed.

There are also controversies in relation to the methods used to evaluate ovarian function. The most commonly used methods are measuring anti-Müllerian hormone (AMH) and follicle-stimulating hormone (FSH) levels and performing antral follicle count (AFC) by ultrasound. Some studies have found AMH to be the most accurate, since this method provides a systemic evaluation with little variation during the menstrual cycle. Other studies have indicated AFC as the best method, since evaluation is performed specifically on the affected ovary, without the effect of any possible compensation by the contralateral ovary. Furthermore, some studies have shown a reduction or no change in AMH, while others have shown no change or even an improvement in AFC following the treatment of endometriomas [40–44].

Another point that has to be taken into consideration is that when endometriomas are compared to cysts of other types, AMH levels seem to be lower in the case of endometriomas even before surgery [27–35]. After surgery, there appears to be a similar decrease, irrespective of the etiology of the cyst [45–47]; however, this decrease is temporary, with a tendency to recover over subsequent months [39, 48–52].

In view of these considerations, the objective of the present study is to evaluate the effect on ovarian function of three different methods of hemostasis (bipolar coagulation versus suture versus hemostatic agents) in patients submitted to oophoroplasty because of an endometrioma, with ovarian function being assessed by measuring AMH and by performing AFC. The use of bipolar energy will be described in detail, including the number of points coagulated during the procedure. To the best of our knowledge, no other study has been conducted to evaluate the three different methods in the same clinical trial.

## Methods

**Fig. 1:**
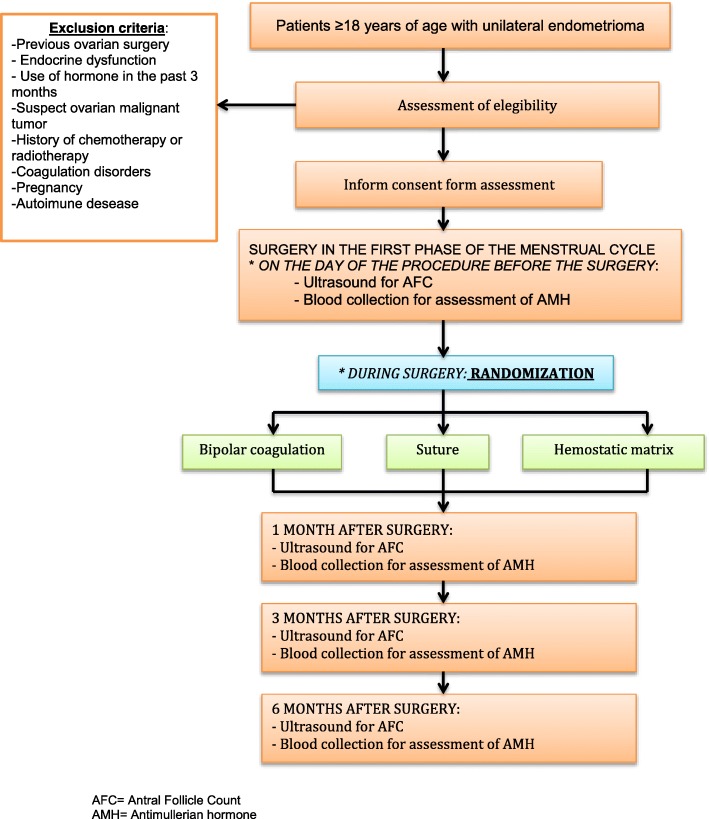
*Flow chart* of the trial

Figure 1 provides an overview of the methodology of the trial. The flow chart will be completed according to the Consolidated Standards of Reporting Trials (CONSORT) 2010 guidelines [53].

### Study design

An open-label RCT will be performed to compare the impact of hemostatic techniques (bipolar coagulation versus laparoscopic suture versus hemostatic matrix) during laparoscopic surgery for an ovarian endometrioma on the ovarian follicular reserve.

### Study population and setting

Patients with ovarian cysts suggestive of endometriomas, diagnosed by ultrasonography, who are receiving care at the gynecological endoscopy outpatient clinic of the Lauro Wanderley University Hospital (HULW) during the study period, will be screened for inclusion in the study.

### Eligibility criteria

Women aged at least 18 years, with regular menstrual cycles (21–35 days) and a diagnosis of a unilateral ovarian cyst suggestive of an endometrioma, with an indication for laparoscopic surgery to remove the cyst due to pelvic pain, infertility, or persistence of the cyst will be eligible for the study. Exclusion criteria are: previous ovarian surgery; endocrine dysfunction (diabetes, thyroid disorders, hyperprolactinemia, adrenal disease, polycystic ovary syndrome); use of hormones in the past three months; suspected malignant ovarian tumor requiring oophorectomy; history of chemotherapy or radiotherapy; coagulation disorders; pregnancy; and autoimmune disease.

### Participant selection and randomization

Eligible patients will be invited to participate in the study and those who accept the invitation will be admitted.

The sample will be divided into three groups according to the hemostatic technique used:

1. Bipolar coagulation (bipolar Maryland, ref.702505ML, Astus Medical©, Copyright 2015, Tampa, FL, USA) with 30-W power and a Valleylab generator (Medronic©, Copyright 2017, Medtronic Parkway, Minneapolis, MN, USA). The number of coagulated points will be counted; the time for coagulation will be measured in seconds.
2. Laparoscopic suturing with simple suture (2–0/Vicrylpolyglactin absorbable synthetic suture; Ethicon Inc., NJ, USA). The number of sutures will be recorded.
3. Hemostatic matrix (Surgicel® Original Absorbable Hemostat, Ethicon, USA).

Randomization to receive the various hemostatic techniques (bipolar coagulation, laparoscopic suture, or hemostatic matrix) during laparoscopic surgery for endometrioma will be performed based on a list of sequential numbers generated using the Random Allocation software package, version 2.0. The list will consist of numbers in the range of 1–84 (the total number of participants to be randomized) and the letters A, B, and C, which will designate the study groups. An individual not directly involved in the study will prepare opaque envelopes numbered 1–84, which will contain the group to which the patient must be allocated.

At the time of inclusion, a number will be assigned to each participant, corresponding to her order of entry in the study. During surgery, at the time of hemostasis, a nurse at the surgical theater will open the envelope containing the corresponding number. Thus, allocation will remain concealed up to that specific moment during surgery, since it is impossible to blind the surgeon to the technique to be used.

### Sample size

Sample size was calculated using resources available at the Laboratory of Epidemiology and Statistics (LEE) website of the Dante Pazzanese Institute (http://www.lee.dante.br/pesquisa/amostragem/qua_2_medias.html). The calculation was based on data provided by Sönmezer et al. [48]. In that article, Sönmezer et al. detected a significant difference in the serum levels of AMH in the first month after surgery, with a measurement of 2.72 ± 1.49 for the patients who received the hemostatic matrix versus 1.64 ± 0.93 for the patients who received bipolar coagulation. Here, we assume that the standard deviation in the AMH measurement of 1.49 in the first month was significant, since that was the number that generated the largest sample size when compared with other values from that article. The difference to be detected is 1.08, which corresponds to the mean difference in AMH in the first month between the patients who receive hemostatic matrix and those who receive bipolar coagulation. On those grounds, and for a statistical power of 80% (*p* = 0.05), each group would have to have 23 participants. Considering possible losses, the sample will be increased by 20% to 28 participants per group, making a total of 84 women. It should be noted that the present sample maybe not detect a powerful difference in treatment arms, but relevant contributions are expected.

### Procedures for the assessment of ovarian reserve

Ovarian reserve will be assessed by measuring AMH and by performing AFC using ultrasonography at four different moments: before surgery and one, three, and six months after surgery. Serum samples will be obtained from each participant and centrifuged for 10 min to separate the cell contents and debris. Each serum sample will be transferred to polypropylene tubes and stored at − 80 °C.

AMH levels will be quantitatively measured using enzyme-linked immunosorbent assay (ELISA) (Diagnostic Systems Laboratories, Webster, TX, USA), with a detection sensitivity of 0.006 ng/mL.

Transvaginal ultrasonography for antral follicle count will be performed during the early proliferative stage (days 3–7 of the menstrual cycle) and the total number of follicles with diameters < 10 mm will be considered.

### Procedures for laparoscopic surgery

The same surgeon will perform all the surgical procedures, with the participants under general anesthesia and in a semi-lithotomy position. A 10-mm umbilical puncture will be performed for the camera after insufflation of the pneumoperitoneum. Three additional 5-mm punctures will be performed on the right iliac fossa, left iliac fossa, and suprapubic area to allow the instruments to be inserted. Intra-abdominal pressure will be maintained at approximately 15 mmHg.

Endometriosis will be classified according to the 1997 Revised American Society for Reproductive Medicine (ASRM) classification. In all the groups, endometriomas will be removed using traction-countertraction techniques. Adhesiolysis will be performed to separate the ovary from the adjacent structures, as necessary. In case of cyst rupture, the contents will be aspirated and the site of spillage will be thoroughly rinsed.

In the group allocated to receive bipolar coagulation, hemostasis will be performed using bipolar tweezers at 30 W as few times as possible, just to control any considerable bleeding. The number of coagulated points will be recorded and the duration of the procedure at each bleeding point will be measured. These parameters will help clarify any possible flaws in previous studies that failed to specify the number of coagulated points or the duration of coagulation at each point.

In the group allocated to receive hemostasis by laparoscopic suture, the procedure will involve simple intra-ovarian sutures (1 or 2 knots with 2–0 Vicryl) and the number of sutures will be counted.

In the group allocated to receive hemostasis by hemostatic matrix, the sealant will be applied on the ovarian wound surface.

### Data collection, processing, and analysis procedures

#### Data collection instrument

**Fig. 2:**
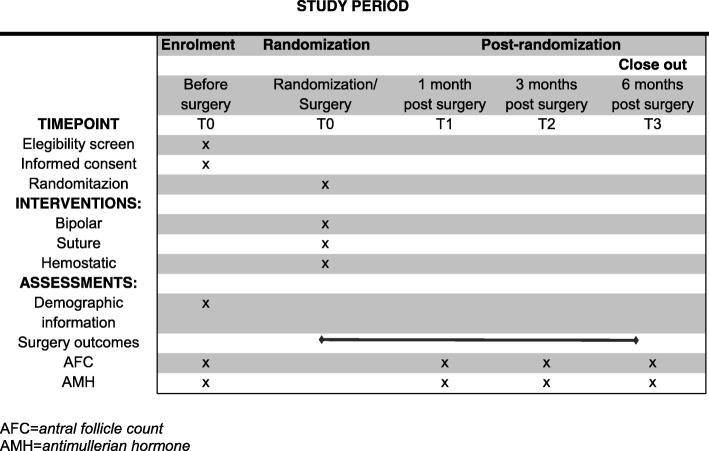
Follow-up items for the participants. Recommendations for Interventional Trials (SPIRIT) figure

A specific form will be developed for the study. It will be in the form of a handwritten log. Prospective data collection will be performed on the day of the intervention and subsequently for the data following the intervention. The follow-up items for the participants are presented in Table 1 and Fig. 2.

**Table 1:** The standard protocol items and timing of the measurements

Measurements	T0	T1	T2	T3
Age, BMI, complaints, gynecological and obstetric history	x			
The largest cyst diameter	x			
Side of pathology	x			
Surgery: duration, blood loss, perioperative complications, ASRM classification	x			
Hemoglobin	x			
Bipolar coagulation (number of points coagulated)	x			
Suture (number of stitches sutured)	x			
Hemostatic (number of hemostatic used)	x			
AMH	x	x	x	x
AFC in affected ovary	x	x	x	x
AFC in the contralateral ovary	x	x	x	x
Total AFC	x	x	x	x

*BMI* body mass index, *T0* before surgery, *T1* one month after surgery, *T2* three months after surgery, *T3* six months after surgery

#### Data processing and analysis

The data will be double-entered by two different data entry operators, using the Epi Info software program, version 7.2. The two databases will be compared using the Validate function of the Epi Info software program. Statistical analysis will be conducted using the Microsoft Excel software and the SPSS statistical software package for Windows, version 19.0.0. The data entry operators and the statistician will be blinded in that they will receive the data marked simply as A, B, or C and will be unaware of which procedure corresponds to which letter. A significance level of 5% will be determined for all the statistical tests.

To analyze the numerical variables AMH and AFC, the Kolmogorov–Smirnov test of normality will be used to determine the type of analysis to be performed. If distribution is normal, two-way analysis of variance (ANOVA) will be used, with the group representing an independent factor (bipolar energy × suture × hemostatic matrix). Another factor to be analyzed will be paired time points (before surgery × 1 month × 3 months × 6 months). If distribution is not normal, non-parametric tests will be used to analyze the variables.

The Kruskal–Wallis test will be used to compare variables between the three groups at each time point, with Tukey’s multiple comparison test being used when comparisons are statistically significant. The Friedman test will be used to perform intra-group comparisons at the four evaluation moments, with Tukey’s multiple comparison test being used when they are significant.

### Ethical issues

The present study complies with the stipulations in the “Declaration of Helsinki” for research involving human beings and with the Brazilian Ministry of Health’s National Health Council Resolution 466/2012 [54]. The internal review board of the Medical Sciences Centre (CCM), Federal University of Paraíba (UFPB) approved the study protocol under reference CAAE 71621717.9.0000.8069, www.plataformabrasil.saude.gov.br.

All of the participants will be informed as to the objectives of the study and will only be included in the study should they agree to participate and sign an informed consent form.

## Discussion

Although laparoscopic cystectomy is the gold standard for the treatment of endometrioma, there are some risks associated with the procedure that may damage ovarian function, even when performed by experienced surgeons [17]. There is considerable controversy in the literature regarding which hemostatic technique causes less damage to the ovarian parenchyma following the stripping of the endometrioma capsule.

To the best of our knowledge, this is the first study to compare three different hemostasis techniques in a single clinical trial, including a detailed assessment of bipolar use. The number of points to be coagulated will be analyzed descriptively, since randomization would risk coagulating more points than required or preventing coagulation when necessary. We believe that the use of bipolar energy may interfere with ovarian function and its judicious use may be associated with less damage; therefore, this variable should be described in detail.

Given the data presently available in the literature, we would like to add to current knowledge by answering the following questions with this RCT:

- Is bipolar energy really responsible for ovarian damage? and
- Is surgical treatment responsible for ovarian damage irrespective of the hemostatic technique used?

Our findings may help surgeons choose the hemostatic method that would cause less damage to ovarian function or, if there are no significant differences between the methods evaluated in this study, surgeons will then be able to choose the most convenient method according to its availability in that particular facility.

### Trial status

Randomization began on 15 March 2018, with 32 patients having been randomized to date (24 April 2019).

## Data Availability

The datasets used and/or analyzed during the current study are available from the corresponding author upon reasonable request.

## Abbreviations

AFC: Antral follicle count
AMH: Anti-Müllerian Hormone
ANOVA: Analysis of variance
ASRM: American Society for Reproductive Medicine
CAAE: Certificate of Presentation for Ethical Assessment
CCM: Medical Sciences Centre
ELISA: Enzyme-linked immunosorbent assay
FSH: Follicle-stimulating hormone
HULW: Lauro Wanderley University Hospital
LEE: Laboratory of Epidemiology and Statistics
RCT: Randomized clinical trial
UFPB: Federal University of Paraíba
USA: United States of America
